# Sources of information influencing decision-making in orthopaedic surgery - an international online survey of 1147 orthopaedic surgeons

**DOI:** 10.1186/1471-2474-14-96

**Published:** 2013-03-14

**Authors:** Arndt P Schulz, Anders Jönsson, Richard Kasch, Prithee Jettoo, Mohit Bhandari

**Affiliations:** 1 University Hospital Lübeck, Lübeck, Germany; 2 Sahlgrenska University Hospital, Göteborg, Sweden; 3 University of Greifswald, Greifswald, Germany; 4 Northern Deanery, Newcastle upon Tyne, UK; 5 McMaster University, Hamilton, Canada; 6 Department of Musculo-Skeletal Surgery, University Hospital Schleswig-Holstein, Campus Lübeck, Ratzeburger Allee 160, Lübeck, 23568, Germany

**Keywords:** Orthopaedics, Survey, Decision-making process, Evidence-based medicine, Online evaluation, Opinion, Internet-based

## Abstract

**Background:**

Manufacturers of implants and materials in the field of orthopaedics use significant amounts of funding to produce informational material to influence the decision-making process of orthopaedic surgeons with regards to choice between novel implants and techniques. It remains unclear how far orthopaedic surgeons are really influenced by the materials supplied by companies or whether other, evidence-based publications have a higher impact on their decision-making. The objective was to evaluate the subjective usefulness and usage of different sources of information upon which orthopaedic surgeons base their decisions when acquiring new implants or techniques.

**Methods:**

We undertook an online survey of 1174 orthopaedic surgeons worldwide (of whom n = 305 were head of their department). The questionnaire included 34 items. Sequences were randomized to reduce possible bias. Questions were closed or semi-open with single or multiple answers. The usage and relevance of different sources of information when learning about and selecting orthopaedic treatments were evaluated. Orthopaedic surgeons and trainees were targeted, and were only allowed to respond once over a period of two weeks. Baseline information included country of workplace, level of experience and orthopaedic subspecialisation. The results were statistically evaluated.

**Results:**

Independent scientific proof had the highest influence on decisions for treatment while OEM (Original Equipment Manufacturer) driven activities like newsletters, white papers or workshops had the least impact. Comparison of answers from the three best-represented countries in this study (Germany, UK and USA) showed some significant differences: Scientific literature and congresses are significantly more important in the US than in the UK or Germany, although they are very important in all countries.

**Conclusions:**

Independent and peer-reviewed sources of information are preferred by surgeons when choosing between methods and implants. Manufacturers of medical devices in orthopaedics employ a considerable workforce to inform or influence hospital managers and leading doctors with marketing activities. Our results indicate that it might be far more effective to channel at least some of these funds into peer-reviewed research projects, thereby assuring significantly higher acceptance of the related products.

## Background

Manufacturers of implants and materials in the field of orthopaedics and traumatology produce a large number of leaflets, white papers and publications each year to influence the decision-making process of orthopaedic surgeons with regards to choice between novel implants and techniques. This puts a large financial burden on these companies [1,2]. On the other hand, the amount of money that companies are willing to spend on clinical research funding and evidence-based research projects appears to be steadily decreasing [3]. Manufacturers often quote compliance rules and internal cost saving regulations as justification. Meanwhile, the same manufacturers are increasingly investing in direct-to-consumer advertising, despite the fact that surgeons feel an overall negative impact on their practice and their interaction with patients [4].

It remains unclear how much orthopaedic surgeons are really influenced by the materials supplied by companies or whether more evidence-based publications have a higher impact on their decision-making. If we could determine whether the latter was a more effective way to guide surgeons, it might be possible to increase the amount of support gained from manufacturers for funding in the area of evidence-based orthopaedics. The funding of research by industry per se is not under public scrutiny, but the fact that sources have not been properly declared in all cases in the past is an issue [5]. Orthopaedic surgeons are increasingly demanding data supporting improvement of expensive new technology and new (and often expensive) treatments [6].

There have been numerous surveys regarding different aspects of decision-making in orthopaedic surgery in the past decades (e.g. [7-14]), and a few of these focused on the decision-making process based on different forms of information [15], mostly related to evidence-based practice [16,17]. It is known that online surveys have a lower response rate than mail questionnaires [17,18], that the response rates to online surveys have been declining since the advent of the internet [19], and that questionnaires should be preceded by a postal letter or pre-contact to increase response rates [19].

On the other hand, there is no proof that increasing the response rate increases study validity in orthopaedics [20]. We therefore decided to use a strictly e-mail and online-based questionnaire without prior postal contact. This approach made it financially possible to contact a large number of surgeons.

The rationale for this study was to gain knowledge about the subjective usefulness and usage of different sources of information upon which orthopaedic surgeons base their decisions when acquiring new implants or techniques.

## Methods

### Study design: internet-based cohort survey

We developed a questionnaire focusing primarily on the factors influencing the deployment of new techniques and implants. Although no patient-related data was used in this study, we asked the local ethical committee to review the study protocol and documentation to assure data safety. After some alterations to the protocol regarding anonymisation and measures against intentional data fraud, the protocol was approved (Ethik-Kommission Lübeck; vote number 10–181). The software keyIngress (Ingress oHG, Norderstedt, Germany) was used for online survey management, including the randomization of items. Reports were generated using the software module keyIngress report from the same company.

**Figure 1:**
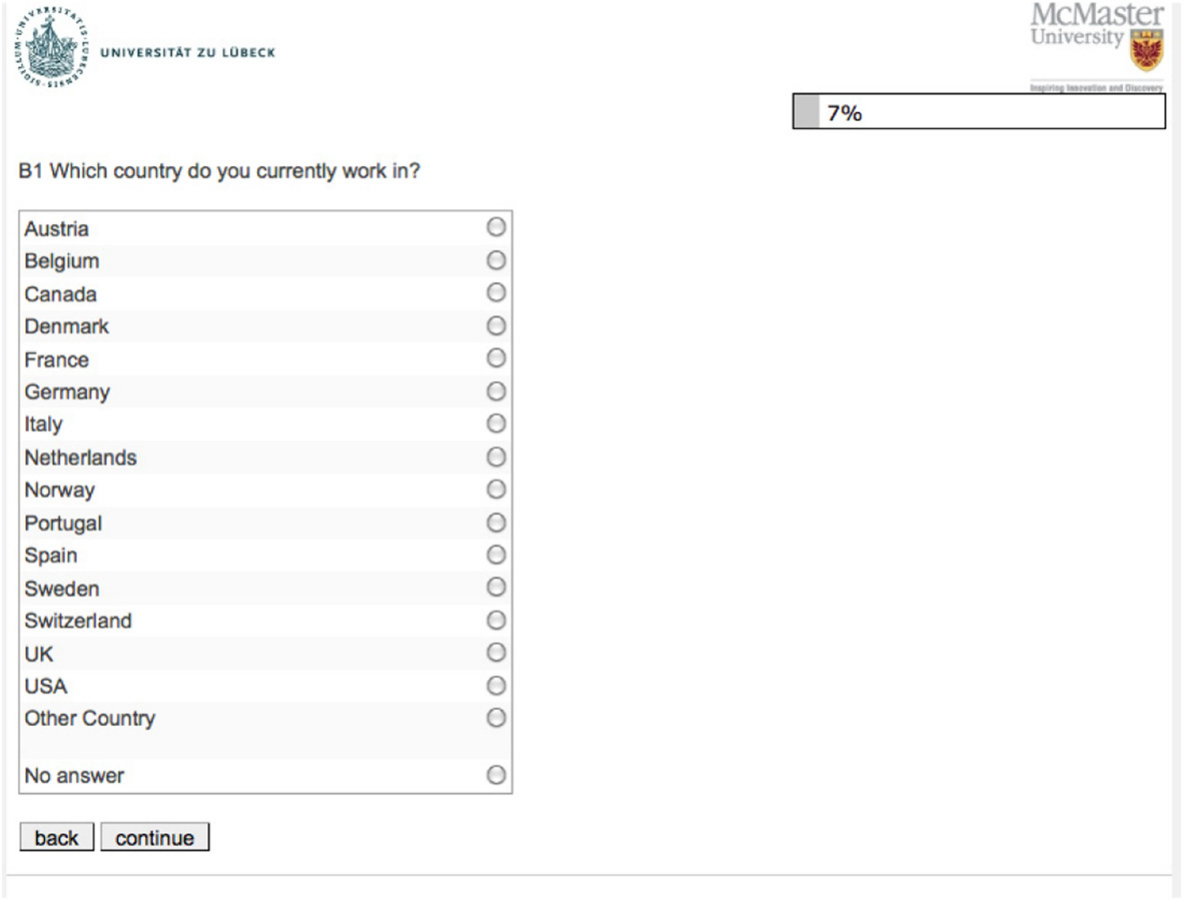
Page one of the survey illustrating the progress bar.

The questionnaire included 34 items. These were provided in blocks of items concerning the same topic, within which the sequence of items was randomized to reduce possible bias by the online survey system. Questions were closed or semi-open with single or multiple answers. A progress bar that stated the percentage of successfully finished pages (14 over all, see Figure 1) was included, and progress to the next page was only possible when all questions had been answered. All questions included the option of a “no answer” reply. A unique link to the questionnaire was provided in the e-mail. The questionnaire could only be filled in once and only within 14 days. After 7 days, a reminder e-mail was sent to all non-responders. All correspondence and the questionnaire were in English. The e-mails were sent out between September 14 and November 18 2010 in three waves.

Overall 12,005 orthopaedic surgeons received an e-mail including a short letter explaining the request to participate in an online survey questionnaire. Contact e-mail addresses were provided by national and international orthopaedic societies and were strictly anonymized. There is thereby no data available about the population initially contacted apart from the fact that all of them were orthopaedic surgeons. This also was the only criterion for eligibility to participate in the study.

The number of fully completed questionnaires and thereby sample size was n = 1174 (for details see Table 1).

**Table 1:** Details of the final study sample size

**Mail-outs and response rates**		
**Total number of email addresses for mail-outs**	**n = 12,005**	
Incorrect email addresses or out-of-office replies	n = 3397	
**Mail-outs sent correctly**	**n = 8608**	100.0%
**Completed questionnaires**	**n = 1174**	**13.6**%
Unfinished questionnaires (incompletely answered)	n = 197	2.2%
Non-responders	n = 7235	

**Figure 2:**
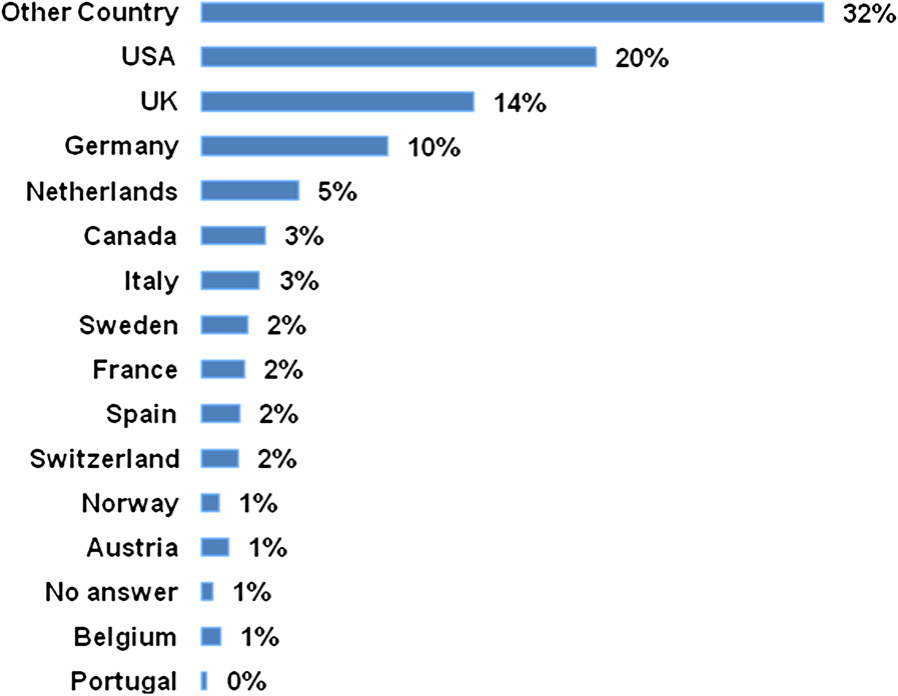
Which country do you work in? (closed, single answer).

Surgeons were asked in which country they were currently working, and not their own nationality. The largest sample stemmed from the United States (20%), followed by the United Kingdom (14%) and Germany (10%) (for details see Figure 2).

Seventy percent of respondents gave their workplace as a teaching hospital or university hospital, 17% were employed in non-academic hospitals and 14% in private practice. For details of the sample see Table 2.

**Table 2:** Description of the sample of 1174 participants

**VALID**	**Frequency**	**Percent**	**Valid percent**	**Cumulative percent**
***Which background does the hospital you are currently working have?***				
**Acadamic**	814	69,3	69,7	69,7
**Non-acadamic**	196	16,7	16,8	86,5
**Private practice**	158	13,5	13,5	100
**Total**	1168	99,5	100	
**Missing**	5	0,4		
**Don’t know**	1	0,1		
**Total**	6	0,5		
**Total**	1174	100		
***What is the level of your experience in orthopaedic surgery?***				
**Junior assistant/registrar (1–3 years experience)**	74	6,3	6,4	6,4
**Senior assistant/registrar (4–6 years experience)**	161	13,7	14	20,5
**Consultant (7–15 years experience)**	463	39,4	40,3	60,7
**Senior consultant (>15 years experience)**	451	38,4	39,3	100
**Total**	1149	97,9	100	
**Missing**	23	2		
**Don’t know**	2	0,2		
**Total**	25	2,1		
**Total**	1174	100		
	***Are you the head of your departmend (chief surgeon)?***				
**Yes**	306	26,1	26,7	26,7	
**No**	841	71,6	73,3	100	
**Total**	1147	97,7	100		
**Missing**	25	2,1			
**Don’t know**	2	0,2			
**Total**	27	2,3			
**Total**	1174	100			

**Figure 3:**
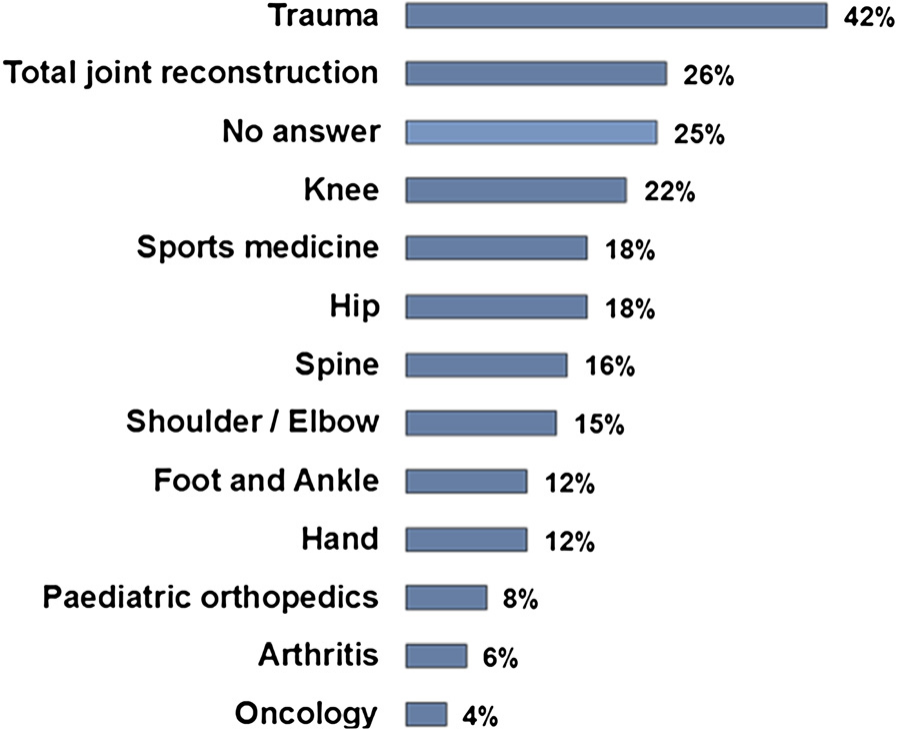
What is your predominant interest in orthopaedics? (semi-open, multiple).

**Figure 4:**
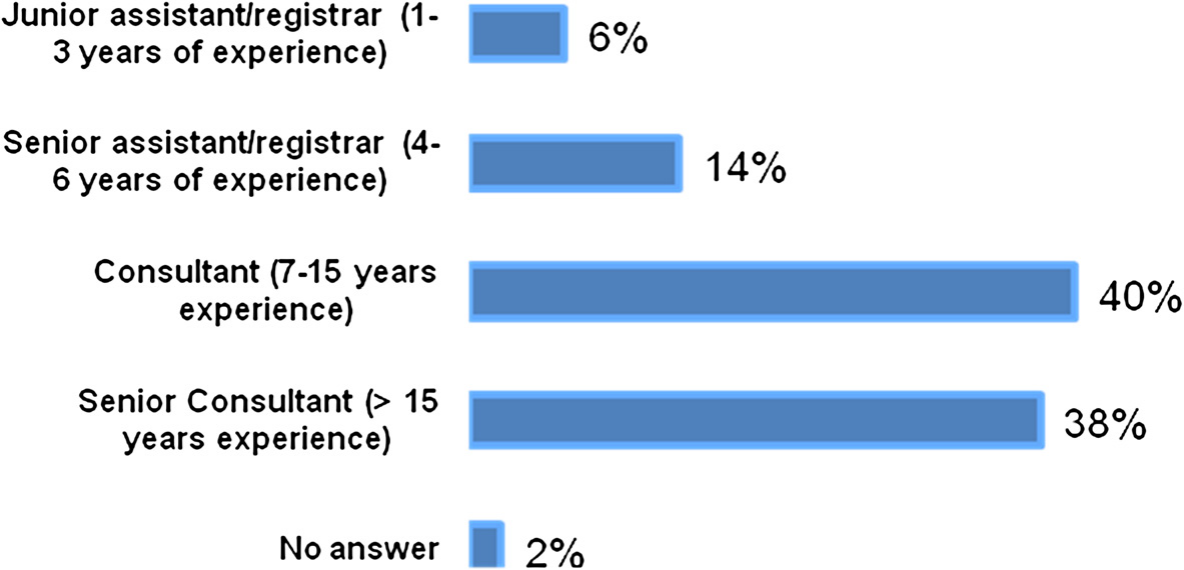
What is the level of your experience in orthopaedic surgery? (closed, single).

The main fields of work were ‘Trauma’ (42%), ‘Total Joint Reconstruction’ (26%) and ‘Knee Surgery’ (22%) (multiple answers, Figure 3). Twenty percent of respondents were undergoing training, and 26% were team leaders or heads of department. Thirty-eight percent of respondents had more than 15 years experience in orthopaedics (see Figure 4). For details of the sample see Table 2.

Statistical analysis was conducted using SPSS (version 19, IBM SPSS, Chicago, USA). Frequency analysis, cross-tabs with bivariate Chi^2^-tests, T-tests for paired samples (repeated measurement) and univariate variance analysis (ANOVA) were employed.

## Results

Overall n = 1174 (13.6% of all successful mail contacts) fully responded to the questionnaire. An incomplete questionnaire was found in n = 197 (2.2%) participants. These had to be excluded according to the study protocol.

The first closed single option question asked how often the different online and print media were read in order to help identify treatments for patients. Scientific literature, e.g. journal article, was read most often with 21% of respondents reading more than 15 articles per month and 14% reading between 11 and 15 articles per month. Newsletters or white papers provided by industrial sources were least frequently read with 56% reading 1 to 5 per month and 31% reading none. There was a statistically significant difference in the average number of journal articles (p = 0.02) and manufacturers’ newsletters (p = 0.01) read between department heads and non-heads with department heads reading more of each source of information.

**Figure 5:**
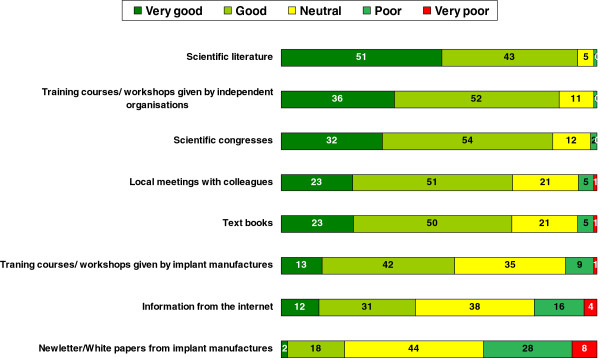
How do you rate the following sources for identifying treatment for your patients? (closed, single, n = 1140–1164, all results in%).

The participants were asked to rate different sources of information with regards choosing a treatment method for patients. Scientific literature, independent training courses and scientific meetings were highly rated. Full details are given in Figure 5. Although the internet was ranked low as a source, 43% of respondents still found it to be a “Good” or “Very Good” way of gaining knowledge. Newsletters and white papers were ranked lowest with the largest group having a “Neutral” position. There were no detectable statistical differences between level of experience or type of hospital.

**Figure 6:**
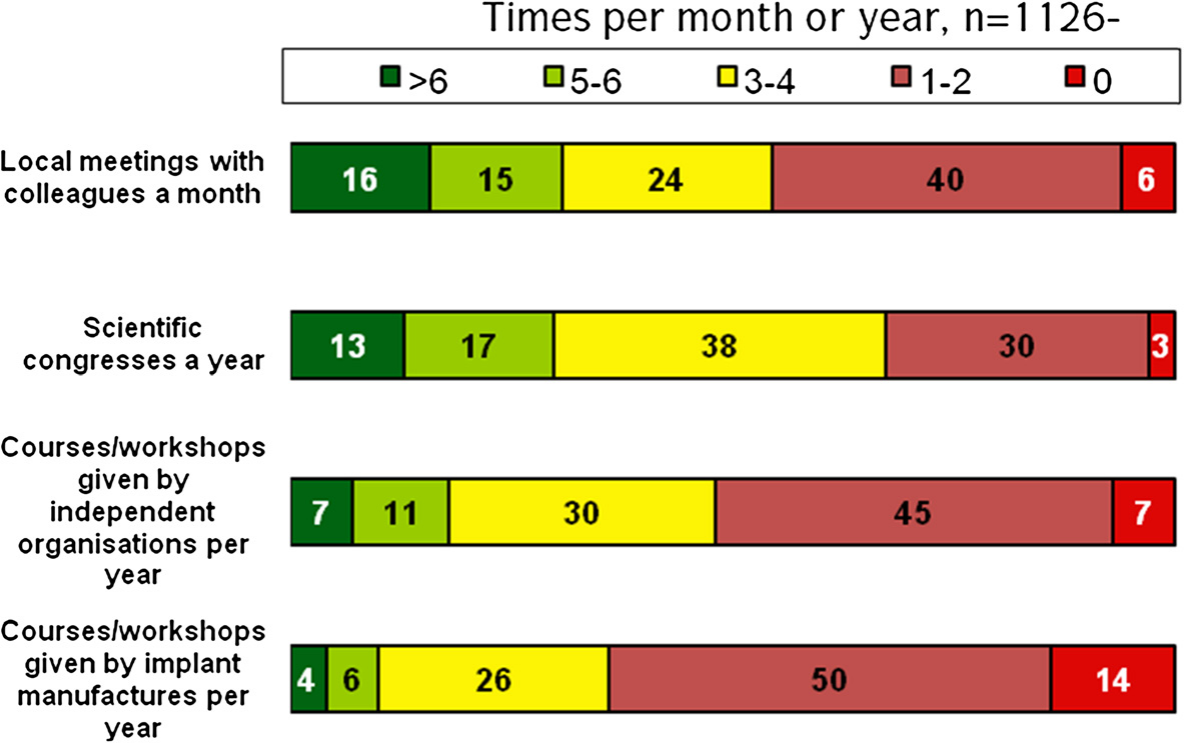
How often do you participate in the following in order to help identify treatment for your patients…? (closed answer, single item).

The next item block enquired about different types of event at which medical knowledge might be gained. The most frequently attended activities were local meetings with colleagues followed by scientific congresses. Training courses or workshops by OEM were frequented regularly by a smaller proportion of respondents (for details see Figure 6). There was no difference between the types of hospital. Department heads attended congresses, courses and workshops significantly more often than other members of staff (p = 0.000 for each item), but there was no difference regarding local meetings.

**Figure 7:**
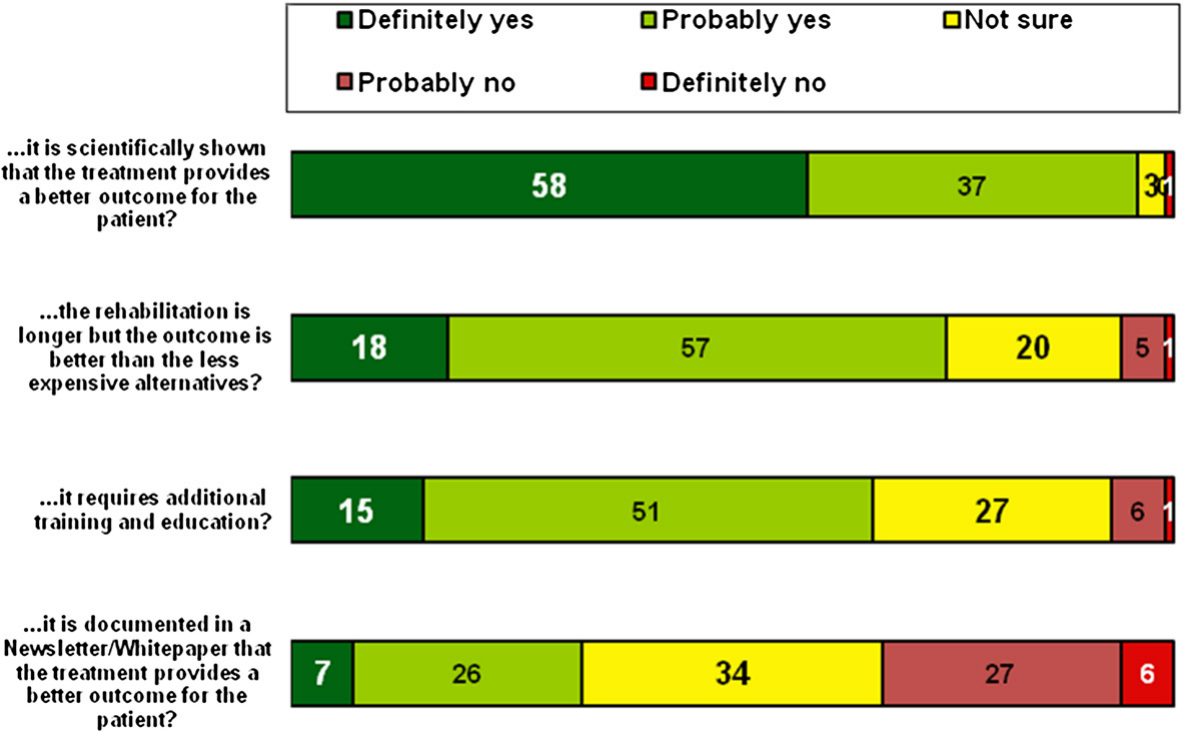
Reasons for a change to a more expensive treatment option; Question: Would you consider offering a more expensive treatment if…? (closed, single, n = 1116–1134, percent).

In the next step, participants were asked which factors would encourage them to choose a more expensive treatment option. The majority would base their decision on scientific evidence of a better outcome. Thirty-three percent stated that they definitely or probably would consider a more expensive treatment option if a company newsletter or white paper stated that the outcome was better (for details see Figure 7). There were no detectable statistical differences regarding type of hospital or level of experience. Statistical differences were found between the three nations with the most participants. Ninety-eight percent of participants from the United States (n = 232) responded to the question ‘would they consider a more expensive treatment if it is scientifically shown to have a better outcome’ with definitely or probably yes compared to just 93% for German participants (n = 106; p = 0.028). Thirty-four percent of participants from the United Kingdom (n = 161) answered the question ‘would they consider a more expensive treatment if the better outcome was documented in a company white paper or newsletter’ with definitely or probably yes compared to just 19% for participants from the United States (p = 0.031). Other trans-national comparisons showed no statistical significant differences.

## Discussion

Online surveys have been successfully used in orthopaedics in recent times either to interview surgeons [21-24] or patients [25]. The response rate of just 13.6% illustrated that it is not easy to persuade a large proportion of surgeons to participate in an online survey. Nevertheless, more than a thousand participants did respond, assuring a good dataset. Furthermore, a low response rate does not necessarily mean low validity [20].

The main aim of our study was to assess the impact of different forms of information in the decision-making process of surgeons. Orthopaedic surgeons are provided with vast amounts of information [26]. Sources like the internet and manufacturers’ leaflets are easy to access and free of charge. Training courses require time and the surgeon incurs costs, a factor that might explain their relatively low usage by junior surgeons [27]. Journal articles also require time to find and study the relevant articles. As scientific literature is often written in English, we cannot exclude a certain language bias.

Nevertheless we clearly found that the sources of information that are preferred by surgeons remain peer-reviewed research articles, presentations at congresses and training courses by independent organisations. Ninety-four percent of respondents judged peer-reviewed scientific sources as a good or very good way of gaining important knowledge in order to identify novel treatment methods. Eighty-seven percent thought the same about independent courses and workshops. Meetings and congresses, as a source of information, were seen as important by 86%. In contrast, manufacturer-initiated publications were only seen by 20% to be a good or very good source. This is in accordance with studies that found evidence-based medicine to be of increasing importance for surgeons. Their impact in terms of changing knowledge, attitudes and uptake in clinical practice has been researched in the past with the aim of improving the use of research findings by individual healthcare professionals [28]. The role of the internet and material provided by manufacturers as a source for decision-making and implementation of new techniques on the other hand has rarely been studied, and even then with suboptimal methodology [29].

One of the limitations of this study is that we did not enquire about which organisations are seen as independent by orthopaedic surgeons. It would be interesting to determine whether organisations that are officially independent but are directly funded by manufacturers are accepted as “independent”. In addition, we did not specify exactly what respondents judged as “information from the internet”.

Statistical evaluation of the three largest participating nations showed small but significant differences that we cannot fully explain. A possible explanation for the relatively high rate of acceptance of “manufacturers’ newsletters and white papers” in the United Kingdom might be attributed to the term “white papers”, which is the name often given to governmental publications or announcements by the national health service [30-33]. This result should be interpreted with caution, as bias cannot therefore be excluded. The language bias is another restriction of our study, we can not be sure that number of participants and results would have been different if the survey would have been multilingual [34,35]. Knobloch described a general tendency to publish positive results in English [36], resulting in a selection bias. The potential limitation of response bias has been widely described [37-42]. In a recent German trial, internet based data sampling showed an additional response bias but had no direct effect on outcome scales [38]. We cannot exclude response bias for our study.

## Conclusions

Manufacturers certainly require large monetary resources each year to plan, create, print and distribute material like websites, newsletters, workshops and white papers. Clearly the aim of these materials is to influence decision-making processes in orthopaedic surgery. The results of our study indicate that it might be far more effective to channel at least some of these funds into peer-reviewed research projects, thereby assuring significantly higher acceptance of their products.

## Competing interests

The authors declare that they have no competing interests.

## Authors’ contributions

APS was involved in the conception and design of the study, data acquisition and drafting the manuscript; AJ was involved in the conception and design of the study and revising the manuscript critically for important intellectual content; RK was involved in evaluation of the data and drafting the manuscript; PJ was involved in evaluation of the data and revising the manuscript critically for important intellectual content; MB was involved in conception and design, statistical evaluation of the data and drafting the manuscript. All authors have given final approval of the version to be published.

## Pre-publication history

The pre-publication history for this paper can be accessed here:

http://www.biomedcentral.com/1471-2474/14/96/prepub
